# Editorial: Advanced technology for human movement rehabilitation and enhancement

**DOI:** 10.3389/fnins.2025.1581451

**Published:** 2025-03-26

**Authors:** Longbin Zhang, Yingbai Hu, Mingming Zhang, Ruoli Wang, Elena M. Gutierrez-Farewik, Wei Tech Ang

**Affiliations:** ^1^School of Robotics, Hunan University, Changsha, China; ^2^School of Mechanical and Aerospace Engineering, Nanyang Technological University, Singapore, Singapore; ^3^Multi-Scale Medical Robotics Centre, The Chinese University of Hong Kong, Shatin, China; ^4^Department of Biomedical Engineering, Southern University of Science and Technology, Shenzhen, China; ^5^KTH MoveAbility Lab, Department of Engineering Mechanics, KTH Royal Institute of Technology, Stockholm, Sweden

**Keywords:** wearable exoskeletons, artificial intelligence, virtual reality, advancing rehabilitation strategies, neuromuscular diseases

In the dynamic field of human movement science, the integration of cutting-edge technology with human physiology is driving a transformative shift in rehabilitation and enhancement. This research theme encompasses neurorehabilitation, assistive robotics, and human-machine interaction, offering exciting prospects for reshaping movement recovery. Translational research plays a key role by bridging scientific advancements with practical clinical applications, ensuring that breakthroughs in laboratories transition seamlessly into real-world treatments. Neurorehabilitation focuses on neuroplasticity and the brain's ability to adapt, while advanced imaging and neurophysiology guide interventions aimed at rewiring neural pathways for individuals with neurological injuries. Assistive robotics combine human potential with sophisticated devices, providing tailored support that aids recovery, enhances muscle activation, joint movement, and gait training, ultimately promoting functional independence. The intersection of human-machine interaction explores the blurring of lines between humans and technology, enabling seamless collaboration to guide and benefit patients through technological assistance.

This Research Topic provides a valuable platform for exploring key mechanisms of human movement rehabilitation, integrating physiological strategies with the design of assistive devices. It highlights the critical role of advanced algorithms, virtual reality, wearable sensors, and machine learning in addressing significant rehabilitation challenges. The studies in this Research Topic cover a broad spectrum of topics, from stroke-induced motor impairments and neurodegenerative conditions to pediatric mobility impairments and sports training. By showcasing groundbreaking technologies, this Research Topic emphasizes the importance of neurophysiological assessments and machine learning models in optimizing rehabilitation outcomes. Examples include motor unit estimation, surface electromyography, and electroencephalogram (EEG)-based movement intention detection to monitor and enhance recovery. Wearable sensors and artificial intelligence technologies are also leveraged to assess gait abnormalities, monitor knee osteoarthritis, and provide real-time feedback in sports training and swimming action recognition. These innovations demonstrate the transformative potential of advanced technology in improving mobility, functional independence, and quality of life for patients across various conditions.

Stroke-induced impairments in gait and upper limb motor function significantly impact mobility, daily activities, and overall quality of life. Pan et al. conducted a case-control study analyzing the biomechanical deviations in gait patterns of post-stroke hemiplegic patients, using statistical parametric mapping to compare joint angles, moments, and ground reaction forces across the gait cycle. Their findings revealed significant abnormalities in both the affected and less-affected limbs, highlighting the need for targeted interventions that address bilateral impairments. Liu et al. conducted a randomized controlled trial comparing robot-assisted therapy (RT) with conventional therapy to enhance upper-limb motor function in post-stroke patients. Their results demonstrated that RT significantly accelerated improvements in Fugl-Meyer Assessment Upper Extremity scores, particularly in patients at Brunnstrom Stage III, although both groups showed similar improvements in functional independence as measured by the Modified Barthel Index. This suggests that robot-assisted therapy holds great potential for enhancing motor recovery in moderate impairment cases. Li et al. examined the predictive value of neuroelectrophysiological assessments, specifically motor unit number estimation (MUNE) and F-waves, for forecasting upper extremity motor function and long-term recovery in stroke patients. They found that MUNE provided more accurate predictions of motor recovery a year post-stroke, emphasizing the importance of incorporating neuroelectrophysiological evaluations into stroke rehabilitation to improve outcomes. Additionally, Lu et al. explored how spasticity confounds myoelectric pattern recognition in stroke rehabilitation. They suggested that botulinum toxin injections could reduce muscle overactivity and improve the effectiveness of myoelectric pattern recognition, supporting the use of this combined approach to enhance rehabilitation outcomes for stroke patients.

The integration of motor unit analysis and muscle synergy research is advancing rehabilitation strategies for neuromuscular diseases and mobility impairments. The loss of motor units in conditions like amyotrophic lateral sclerosis and muscular dystrophy leads to muscle weakness and atrophy, making tools like motor unit number estimation and compound muscle action potential (CMAP) valuable for tracking motor unit loss and compensatory reinnervation. Zhang D. et al. studied the impact of electrode recording areas on CMAP parameters, discovering that electrode size did not significantly affect CMAP data, which enhances the interpretation of CMAP scan results in rehabilitation contexts. Tsuchiya et al. explored how pedaling speed and direction influence the recruitment of muscle synergies, showing that specific muscle groups are recruited depending on the conditions, such as quadriceps and hip extensors at 30 revolutions per minute (RPM) and hamstring-dominant synergies at 60 RPM during forward pedaling. Their findings suggest that pedaling exercises tailored to different speeds and directions can better target specific muscles for rehabilitation, particularly for stroke patients and those with mobility impairments. These studies highlight the growing potential for personalized rehabilitation strategies that optimize recovery by considering individual muscle synergies and motor patterns.

Wearable robotic exoskeletons are increasingly recognized for their potential to assist rehabilitation in conditions like stroke-induced drop foot and pediatric mobility impairments such as cerebral palsy. Zhang X. et al. developed a soft ankle exoskeleton to assist with dorsiflexion and eversion, demonstrating its ability to significantly improve gait kinematics in both seated and gait tests with minimal errors. Bradley et al. assessed the clinical impact of the pediatric Trexo Plus exoskeleton, focusing on motor, neural, and muscular outcomes in children with severe mobility impairments. Their feasibility study highlighted the exoskeleton's potential to promote neuroplasticity and improve mobility, particularly in young patients with motor impairments. Miao et al. proposed a framework for robot-assisted upper-limb rehabilitation, using subject-specific workspace constraints and performance-based control strategies to enhance training effectiveness, natural motion, and patient engagement. Voß et al. emphasized the importance of intuitive control architectures for lower-limb prostheses, combining volitional control with proprioceptive feedback to improve user satisfaction and functionality. These studies demonstrate the promising potential of exoskeletons and advanced control systems to revolutionize rehabilitation, offering significant improvements across diverse patient populations.

Artificial intelligence and sensor technologies are at the forefront of human movement rehabilitation and performance enhancement, driving advancements in precision feedback and personalized interventions. LinLin et al. developed a system that combines Vision Transformers with Contrastive Language-Image Pretraining (CLIP) models to deliver real-time, context-aware feedback in sports training, outperforming traditional methods in accuracy, recall, and computational efficiency. Chen and Yue introduced Swimtrans Net, which uses Swin-Transformer and CLIP models to improve swimming action recognition and provide real-time feedback with exceptional accuracy. Sarmah et al. explored a non-invasive method for detecting knee osteoarthritis (Knee OA) and monitoring rehabilitation progress by integrating gait and muscle activity data with machine learning models. Their work demonstrated how wearable sensors and non-knee joint variables could be leveraged for early Knee OA detection and personalized interventions, illustrating the potential of artificial intelligence and sensor technologies to revolutionize both rehabilitation and sports performance through precision feedback and tailored solutions.

Virtual reality (VR)-based interventions are emerging as powerful tools in rehabilitation, offering cost-effective solutions for home-based recovery. Dai et al. developed a VR-based rehabilitation system that reconstructs full-body poses from sparse motion signals, providing real-time motion correction and allowing patients to undergo efficient therapy without frequent clinical visits. Dong et al. enhanced movement intention detection for neurological rehabilitation by using VR induction to improve EEG signal detectability, facilitating better activation of brain functional networks. These innovations highlight the growing role of VR in rehabilitation, demonstrating its potential to enhance motor recovery, engagement, and accessibility.

This Research Topic highlights the important role of advanced technologies in advancing human movement rehabilitation and enhancement. By exploring the intersection of assistive robotics, virtual reality, wearable sensors, and AI-powered systems, the research illustrates how engineering and human physiology work together to address a wide range of challenges in mobility and rehabilitation. Through a thoughtful integration of application, this body of work underscores the potential for impactful interventions that could contribute to the progression of rehabilitation science. By connecting theoretical advancements with practical implementations, these studies present opportunities to develop more personalized, accessible, and effective treatments for patients globally, shaping the future direction of rehabilitation.

